# Sucrose-induced stomatal closure is conserved across evolution

**DOI:** 10.1371/journal.pone.0205359

**Published:** 2018-10-12

**Authors:** Jayaram Kottapalli, Rakefet David-Schwartz, Belal Khamaisi, Danja Brandsma, Nitsan Lugassi, Aiman Egbaria, Gilor Kelly, David Granot

**Affiliations:** Institute of Plant Sciences, Agricultural Research Organization, The Volcani Center, Rishon Le-Zion, Israel; University College Dublin, IRELAND

## Abstract

As plants evolved to function on land, they developed stomata for effective gas exchange, for photosynthesis and for controlling water loss. We have recently shown that sugars, as the end product of photosynthesis, close the stomata of various angiosperm species, to coordinate sugar production with water loss. In the current study, we examined the sugar responses of the stomata of phylogenetically different plant species and species that employ different photosynthetic mechanisms (i.e., C_3_, C_4_ and CAM). To examine the effect of sucrose on stomata, we treated leaves with sucrose and then measured their stomatal apertures. Sucrose reduced stomatal aperture, as compared to an osmotic control, suggesting that regulation of stomata by sugars is a trait that evolved early in evolutionary history and has been conserved across different groups of plants.

## Introduction

The conquest of land by plants was an evolutionary process that began more than 450 million years ago [1]. Fossil, phylogenetic and molecular records indicate that the green algae and stoneworts (Charales) are probably the extant origin group of all land plants [2]. Extant land plants include three ‘non-vascular’ lineages, the liverworts, mosses and hornworts, collectively known as bryophytes. The remaining extant land plants are known as vascular plants (i.e., pteridophytes, gymnosperms and angiosperms), as they all possess complex water-conducting xylem tissue.

The terrestrial ecosystem posed numerous challenges to plants with aquatic ancestry, including drought. To survive in the terrestrial environment, early plants evolved an impermeable outer cuticle that prevents desiccation, but also restricts the direct exchange of gases with the surroundings, limiting the entry of CO_2_ required for photosynthesis [3]. To facilitate gas exchange, land plants developed microscopic pores called stomata on their outer surface. These can be seen in the mosses and hornworts and in the subsequent evolutionary vascular lineages [4], although the stomata of hornworts and mosses might facilitate desiccation of spore capsules [5].

Stomata are made up of two guard cells that can swell or shrink. Swelling opens the stomata and shrinking closes the stomata. This swelling and shrinking is caused by turgor changes within the guard cells. The movement of water into the guard cells increases their turgor, causing the guard cells to swell and opening the stomata. Movement of water out of the guard cells reduces their turgor, causing the guard cells to shrink and closing the stomata. Changes in guard-cell turgor occur in response to different environmental and physiological conditions. Those turgor changes may be caused by passive changes in guard-cell water content due to hydration or dehydration of the epidermis and guard cells, or by active processes that increase or decrease the levels of guard-cell osmolytes. Increased osmolyte content drives water into the guard cells, opening the stomata, and decreased osmolyte content drives water out of the guard cells, closing the stomata. Light stimulates stomatal opening in many plant species and active stomatal opening in response to light was probably a trait of the earliest bryophytes [6]. Active stomatal closure due to reduced osmolyte content of guard cells, which decreases the turgor of those cells causing the stomata to close, evolved in gymnosperms and angiosperms. However, the existence of this mechanism in the earliest evolutionary lineages is still a matter of debate [7, 8].

It was long thought that sugars are the primary osmolytes that accumulate in guard cells at dawn to open stomata [9]. That theory was later replaced by the discovery that potassium ions, together with malate and chloride counter-ions, are the primary osmolytes that open stomata [10–14]. Yet, based on the correlations between the ion content of guard cells, their sugar content and stomatal aperture over the course of the day, it has been suggested that potassium ions open stomata at dawn and that sucrose replaces those potassium ions in the middle of the day, taking up the role of the osmolyte that keeps the stomata open [15]. Other studies have found that over the course of the photoperiod, the concentration of sucrose in the leaf apoplast of *Vicia faba* increases up to 2 mM and that some of that sucrose is carried by the transpiration stream toward the guard cells and accumulates outside the guard cells to reach a concentration of up to 150 mM, perhaps imposing an extracellular osmotic effect that drives water out of the guard cells and closes the stomata [16–20]. However, recent discoveries have shown that sucrose, the primary end product of photosynthesis, does indeed close rather than open the stomata of Arabidopsis, tomato (*Solanum lycopersicum*) and *V*. *faba*, but that occurs independent of any extracellular osmotic effect [21–24]. Rather, stomatal closure by sucrose is mediated by hexokinase (HXK) within guard cells, a well characterized sugar sensor [21, 23, 25]. Based on these observations, it has been suggested that sugar-sensing within guard cells is a feedback mechanism that coordinates photosynthesis with the rate of transpiration [21].

According to this hypothesis, when sugar production exceeds phloem-loading and translocation capacity, more sugars might be carried toward the guard cells, enter those cells and be sensed within guard cells, stimulating stomatal closure [21, 26, 27]. Sucrose may enter the guard cells via a sucrose transporter and then be cleaved within the guard cells, or cleaved by apoplastic invertase to glucose and fructose, which may then enter the guard cells via hexose transporters; all of these transporters have been identified in guard cells [28–30]. The hexose monomers (glucose and fructose) are substrates of HXK and as such can be sensed by HXK within guard cells and stimulate stomatal closure, forming a feedback mechanism that coordinates sugar production with transpiration, to reduce water loss [21, 25].

The sucrose-induced stomatal closure effect has, to date, been observed in a few angiosperms: Arabidopsis, tomato and *V*. *faba* [21–24]. In the current study, we examined the sugar response of stomata in different angiosperm species, including species that use different photosynthetic mechanisms (i.e., C_3_, C_4_ and CAM).

## Materials and methods

### Plant material

The plant material used in this study was grown under semi-controlled greenhouse conditions or under natural (ambient light) conditions. Plant material was collected at the ARO Center (http://www.agri.gov.il) and the botanical garden of Tel Aviv University (http://botanic.tau.ac.il). The plant species used in this study are listed in S1 Table.

### Stomatal measurements

Stomatal aperture was determined using the rapid imprinting technique described by Geisler and Sack [31] with minor modifications [21]. In brief, light-bodied vinylpolysiloxane dental resin (Heraeus-Kulzer, Hanau, Germany) was attached to the abaxial leaf side and then removed as soon as it had dried (approx. 30 s to 1 min). The resin epidermal imprints were covered with nail polish, which was removed once it had dried and served as a mirror image of the resin imprint. The nail-polish imprints were transferred to glass slides and photographed under a bright-field inverted microscope [Leica DMLB epi-fluorescence microscope (Leica Microsystems, Wetzlar, Germany) with Nikon DS-Fi1 digital camera using NIS-Elements BR 3.0 software (Japan)]. Stomatal images were later analyzed to determine aperture size using the ImageJ software (http://rsb.info.nih.gov/ij/) fit-ellipse tool.

To asses stomatal response, leaves / leaflets (including the petiole, if possible), were cut and immediately immersed in artificial xylem sap solution (AS, control) [32] or AS containing sucrose (Duchefa Biochemie) or sorbitol (Sigma-Aldrich). The sorbitol treatment served as a non-metabolic osmotic control. For plants with too wide or elongated leaves, pieces of leaf were cut and immersed in the above mentioned solutions. Imprints were taken 3 h after immersion and stomatal aperture was analyzed. Experiments were conducted 2 h after sunrise and were carried out for 3 h between 9:00 a.m. to 12:00 p.m., with the exception of *B*. *daigremontianum*, a CAM (Crassulacean Acid Metabolism) plant with stomata that are open at night, for which experiments were carried out between 18:00 and 20:30. Each experiment was repeated two to three times with similar results.

### Stomatal reactivity to sucrose and minimal-water-requirement data

Stomatal reactivity to sucrose (percentage) was calculated as 1 - (aperture _sucrose_ / aperture _sorbitol_); the ratio between stomatal aperture following treatment with sucrose, divided by the aperture following treatment with sorbitol. The minimal water requirement was defined as the minimal amount of water needed to complete a full season of growth, from germination to yield production. Minimal-water-requirement data for the different species used in this study were obtained from previous publications and public databases, as follows: *Populus angulate* [33], *Ricinus communis* (https://www.cabi.org/isc/datasheet/47618), *Catharanthus roseus* (https://www.cabi.org/isc/datasheet/16884), *Moringa oleifera* [34], *Vitis vinifera* [35], *Melia azadirachta* [36], *Triticum aestivum* [37], *Zea mays* [38], *Sorghum bicolor* [39], *Oxalis corniculata* (http://www.herbiguide.com.au/Descriptions/hg_Soursob.htm), *Citrullus lanatus* [40], *Cucumis melo* [41], *Cucurbita pepo* [42] and *Ocimum basilicum* [43].

### Statistical analysis

The statistical analysis was performed using the JMP 5.0 software program. Means were compared using Tukey's HSD. Means were considered to be significantly different at *P* < 0.05. The number of biological repeats and number of stomata analyzed for each species are listed in S1 Table.

## Results

### Stomatal response to sucrose among eudicots and monocots

**Fig 1:**
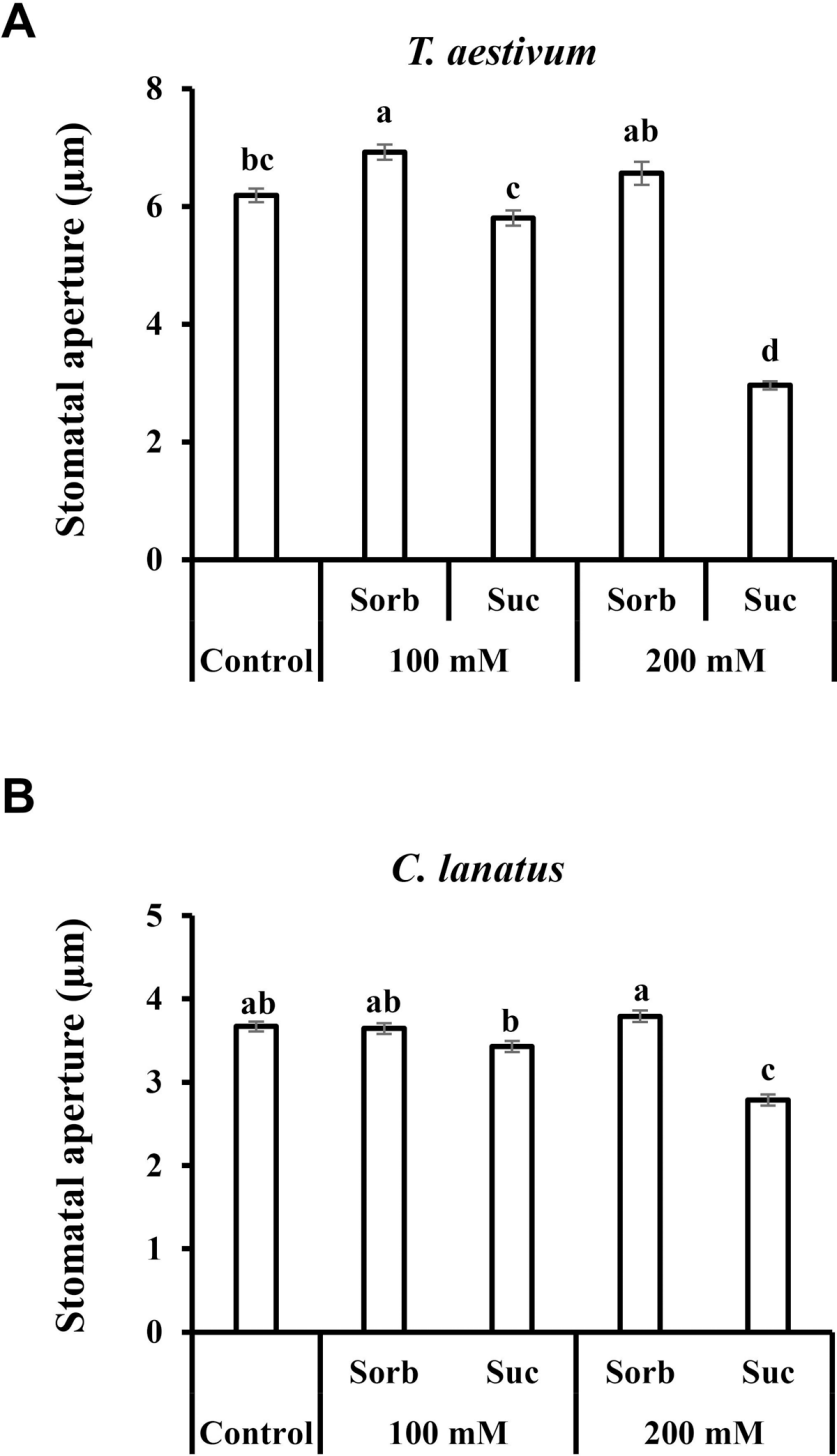
Sucrose-induced stomatal closure in wheat and watermelon. Stomatal response to sucrose in **A**) *T*. *aestivum* and **B**) *C*. *lanatus*. This response was assayed by immersing detached leaves for 3 h in artificial apoplastic sap (AS, control) or AS containing 100 mM or 200 mM sorbitol (as an osmotic control) or 100 mM or 200 mM Suc. Data points are means ± SE. [The numbers of independent biological repeats and stomata (*n*) analyzed for each species are listed in S1 Table.] Different letters indicate a significant difference (Tukey's HSD test, *P* < 0.05).

The effect of sucrose on stomatal aperture was examined among a diverse collection of 20 species of vascular plants by immersing leaves in artificial xylem (apoplastic) solutions (AS) [32] with and without 200 mM sucrose (Suc), about the concentration (150 mM) previously measured around guard cells of *V*. *faba* during the photoperiod [20]. To distinguish between osmotic and a non-osmotic effects, we used sorbitol as an osmotic control. The decision to use a concentration of 200 mM was based on several previous observations: Medeiros [24] observed stronger closure of Arabidopsis stomata by 100 mM sucrose than by 10 mM and, in a previous study, we observed stronger closure of tomato stomata by 200 mM glucose than by 100 mM glucose [21]. We also examined the stomatal responses of two other species belonging to different families, wheat (*Triticum aestivum*) and watermelon (*Citrullus lanatus*), to 100 mM and 200 mM sucrose (Fig 1). In both of those species, the stomatal closure by 200 mM sucrose was stronger than that triggered by 100 mM sucrose, and the closure induced by 100 mM sucrose was significantly stronger than that triggered by 200 mM sorbitol (the osmotic control), indicating that sucrose closes stomata regardless of any increase in the osmolarity of the external solution that could potentially arise from cleavage of sucrose by apoplastic invertase into glucose and fructose. This conclusion is corroborated by previous results obtained with Arabidopsis and tomato plants. In Arabidopsis, it was shown that 10 mM of sucrose stimulates stomatal closure more strongly than 100 mM mannitol, the osmotic control [24]. In tomato it was shown that 100 mM sucrose stimulates stomatal closure more strongly than 200 mM mannitol, the osmotic control [21], eliminating the possibility that stomatal closure by sucrose occurs due to increased osmolarity from sucrose cleavage. Due to the stronger stomatal closure induced by 200 mM sucrose and due to the previous report of a 150 mM concentration of sucrose in the apoplast of *V*. *faba* guard cells [20], we chose 200 mM as a standard concentration for our assays. Using a standard concentration also allowed us to compare the stomatal response across various species.

**Fig 2:**
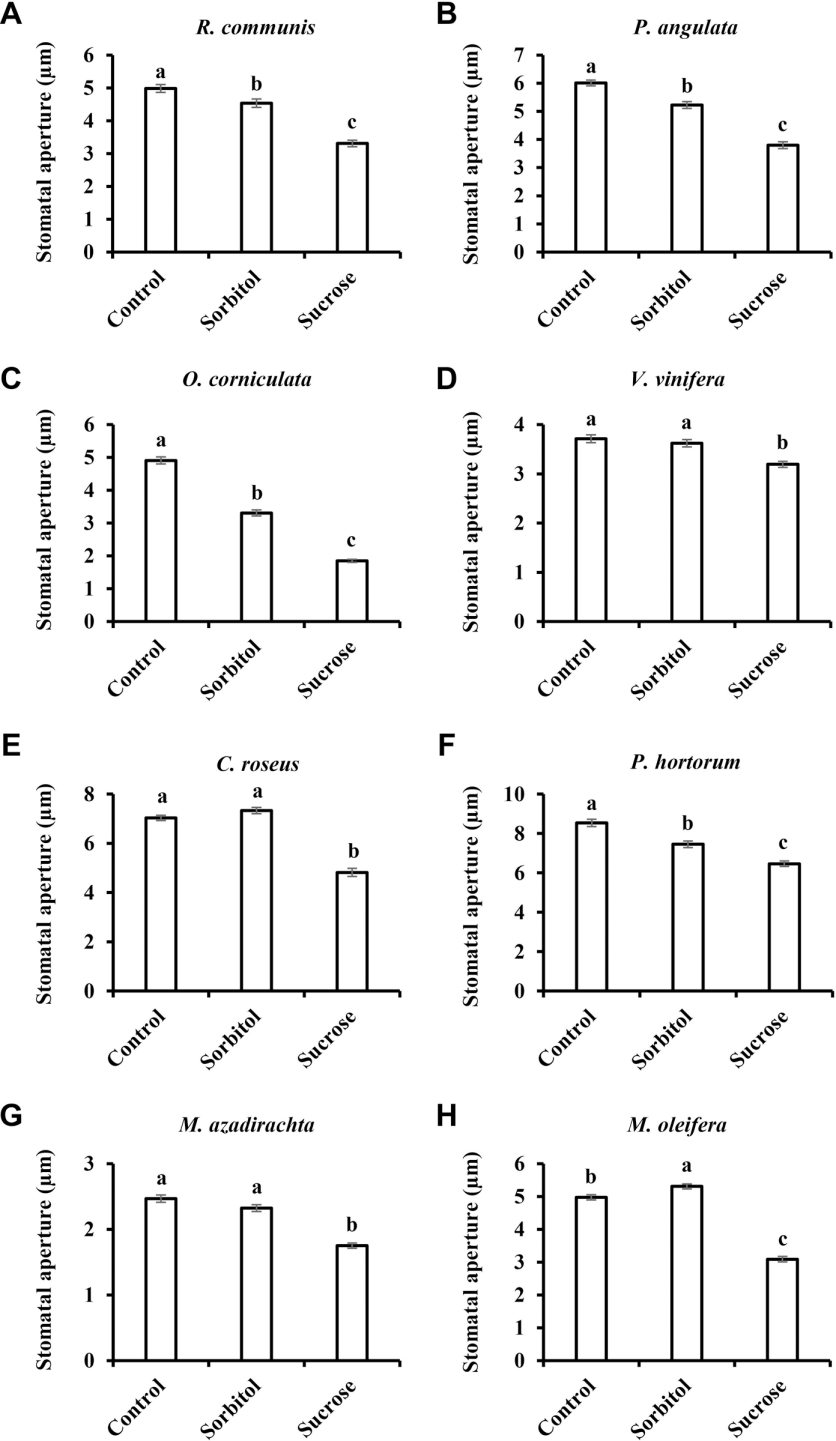
Sucrose-induced stomatal closure in eudicots. Stomatal response to sucrose in the eudicots **A**) *R*. *communis*, **B**) *P*. *angulata*, **C**) *O*. *corniculata*, **D**) *V*. *vinifera*, **E**) *C*. *roseus*, **F**) *P*. *hortorum*, **G**) *M*. *azadirachta* and **H**) *M*. *oleifera*. This response was assayed by immersing detached leaves for 3 h in artificial apoplastic sap (AS, control) or AS containing 200 mM sorbitol (as an osmotic control) or 200 mM Suc. Data points are means ± SE. [The numbers of independent biological repeats and stomata (*n*) analyzed for each species are listed in S1 Table.] Different letters indicate a significant difference (Tukey's HSD test, *P* < 0.05).

Following the sucrose-induced stomatal closure observed in wheat and watermelon (54% and 26% relative to the osmotic control, respectively; Fig 1), we examined additional species belonging to eight different families of the eudicot class of angiosperms. We tested the stomatal response to Suc in *Ricinus communis* (Euphorbiaceae), *Populus angulata* (Salicaceae), *Oxalis corniculata* (Oxalidaceae), *Vitis vinifera* (Vitaceae), *Catharanthus roseus* (Apocyanaceae), *Pelargonium hortorum* (Geraniaceae), *Melia azadirachta* (Meliaceae) and *Moringa oleifera* (Moringaceae) (Fig 2). Suc decreased stomatal aperture size relative to the osmotic control in all eight of these species (Fig 2). While a mild closure response was noted for *V*. *vinifera* (12%) and *P*. *hortorum* (13%), more intense responses were documented for *R*. *communis*, *P*. *angulate* and *M*. *azadirachta* (27%, 27% and 25%, respectively). Out of the eight eudicot species examined, three displayed an even stronger response; from 34% in *C*. *roseus* to 42% and 44% in *M*. *oleifera* and *O*. *corniculata*, respectively (Fig 2). In a few cases, reduced stomatal apertures were also observed for the osmotic control, perhaps due to an extracellular osmotic effect (Fig 2A–2C and 2F). In *M*. *oleifera* the osmotic control sorbitol had a slight opening effect (Fig 2H). However, in all cases, Suc had a stronger closure effect when compared to the osmotic control, supporting an osmotic-independent role for Suc in the regulation of stomatal closure, a response that varies in its intensity among different species.

**Fig 3:**
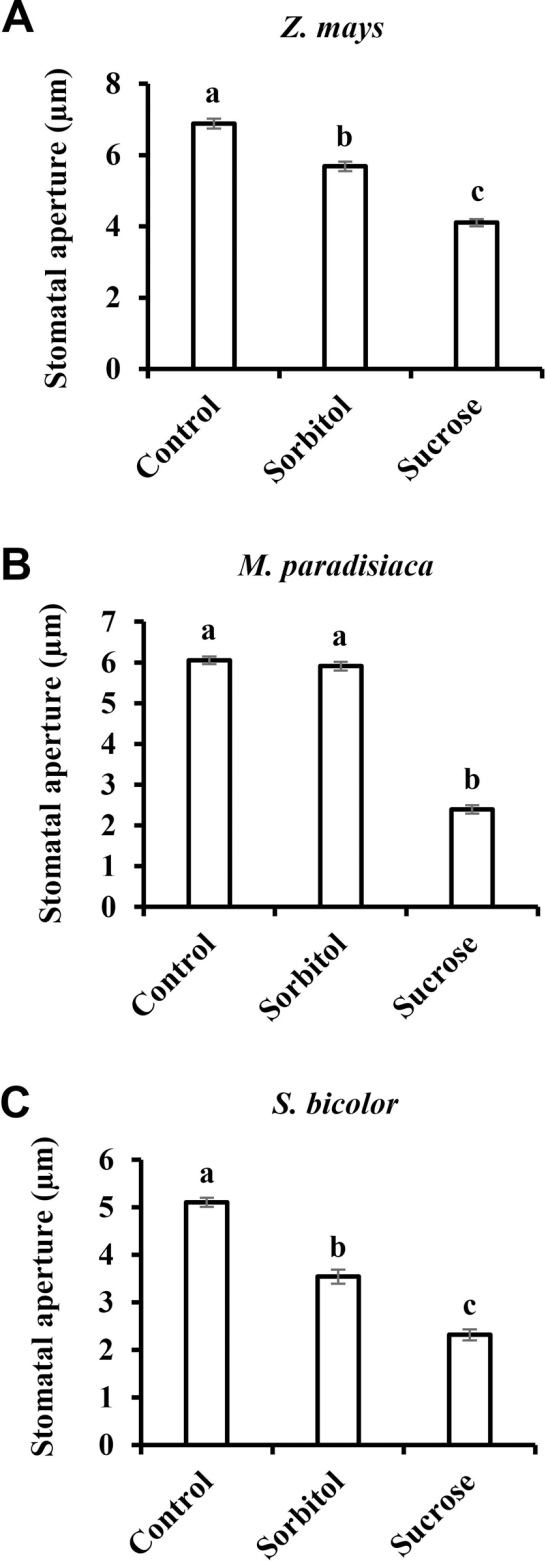
Sucrose-induced stomatal closure in monocots. Stomatal response to sucrose in the monocots **A**) *Z*. *mays*, **B**) *M*. *paradisiaca* and **C**) *S*. *bicolor*. This response was assayed by immersing detached leaves for 3 h in artificial apoplastic sap (AS, control) or AS containing 200 mM sorbitol (as an osmotic control) or 200 mM Suc. Data points are means ± SE. [The numbers of independent biological repeats and stomata (*n*) analyzed for each species are listed in S1 Table.] Different letters indicate a significant difference (Tukey's HSD test, *P* < 0.05).

Similar results were obtained when we tested the stomatal response to Suc among species belonging to the Monocot class of angiosperms. Sucrose stimulated stomatal closure relative to the osmotic control in *Triticum aestivum*, *Zea mays*, *Musa paradisiaca* and *Sorghum bicolor* by 54%, 28%, 59% and35%, respectively (Figs 1A and 3). It appears that in monocots, the stomatal response to Suc is stronger than that seen in eudicots. Yet, both eudicots and monocots share a similar pattern of response to Suc (Figs 2 and 3).

### Stomatal response to sucrose with respect to the phloem-loading pathway

**Fig 4:**
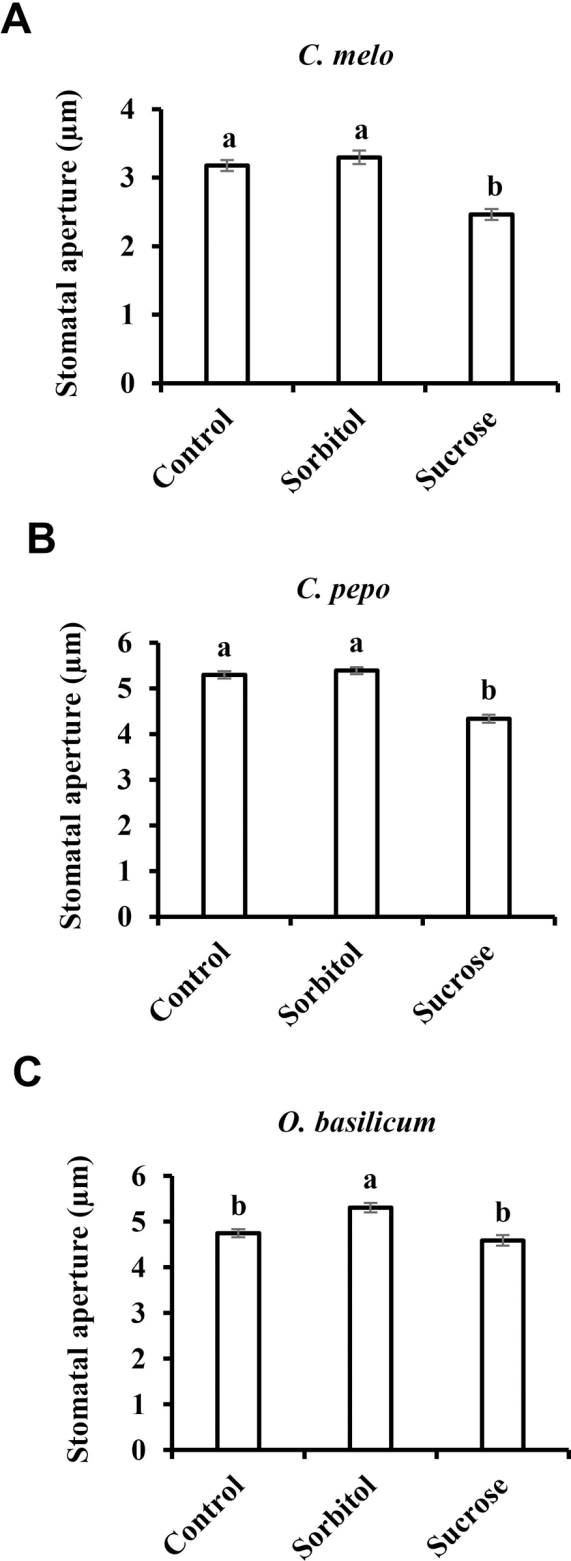
Stomatal response to sucrose in symplastic loaders. Stomatal response to sucrose in the symplastic phloem-loading plants **A**) *C*. *melo*, **B**) *C*. *pepo* and **C**) *O*. *basilicum*. This response was assayed by immersing detached leaves for 3 h in artificial apoplastic sap (AS, control) or AS containing 200 mM sorbitol (as an osmotic control) or 200 mM Suc. Data points are means ± SE. [The numbers of independent biological repeats and stomata (*n*) analyzed for each species are listed in S1 Table.] Different letters indicate a significant difference (Tukey's HSD test, *P* < 0.05).

The movement of sugar from photosynthetic cells to the phloem may occur apoplastically or symplastically [44]. Apoplastic transport involves the movement of sugar from the mesophyll cells into the intracellular space and subsequent loading into the phloem sieve elements. Symplastic transport involves the movement of sugar via plasmodesmata into the companion-cell sieve element complex in the phloem [44]. It has been suggested that the accumulation of sugar in the guard cells’ apoplast corresponds to an apoplastic phloem-loading mechanism, so that sugars carried by the transpiration stream accumulate in significant amounts near the guard cells of apoplastic-loading plants; whereas the concentrations of sugar in the leaves and around the guard cells of symplastic loaders are lower than those seen in apoplastic loaders [45]. Nonetheless, sugars, including sucrose, are found in the apoplast of symplastic loaders [46–49] and, therefore, may also stimulate stomatal closure. To test the effect of sucrose on symplastic loaders, we examined Cucurbitaceae species and basil (*Ocimum basilicum*) to see how their stomata respond to sucrose. The stomatal apertures of *Citrullus lanatus*, *Cucumis melo Cucurbita pepo* and *O*. *basilicum* decreased in size by 26%, 25%, 19% and 13%, respectively, when they were treated with sucrose instead of the osmotic control (Figs 1B, 4A and 4B). Yet, in *O*. *basilicum* sorbitol had a slight opening effect, and stomatal closure was observed only relative to sorbitol and not relative to the non-osmotic control (Fig 4C). With the possible exception of the data collected in basil, these results suggest that the stomatal response to Suc also occurs in symplastic phloem-loading species.

### Stomatal response to sucrose with respect to photosynthetic strategy

**Fig 5:**
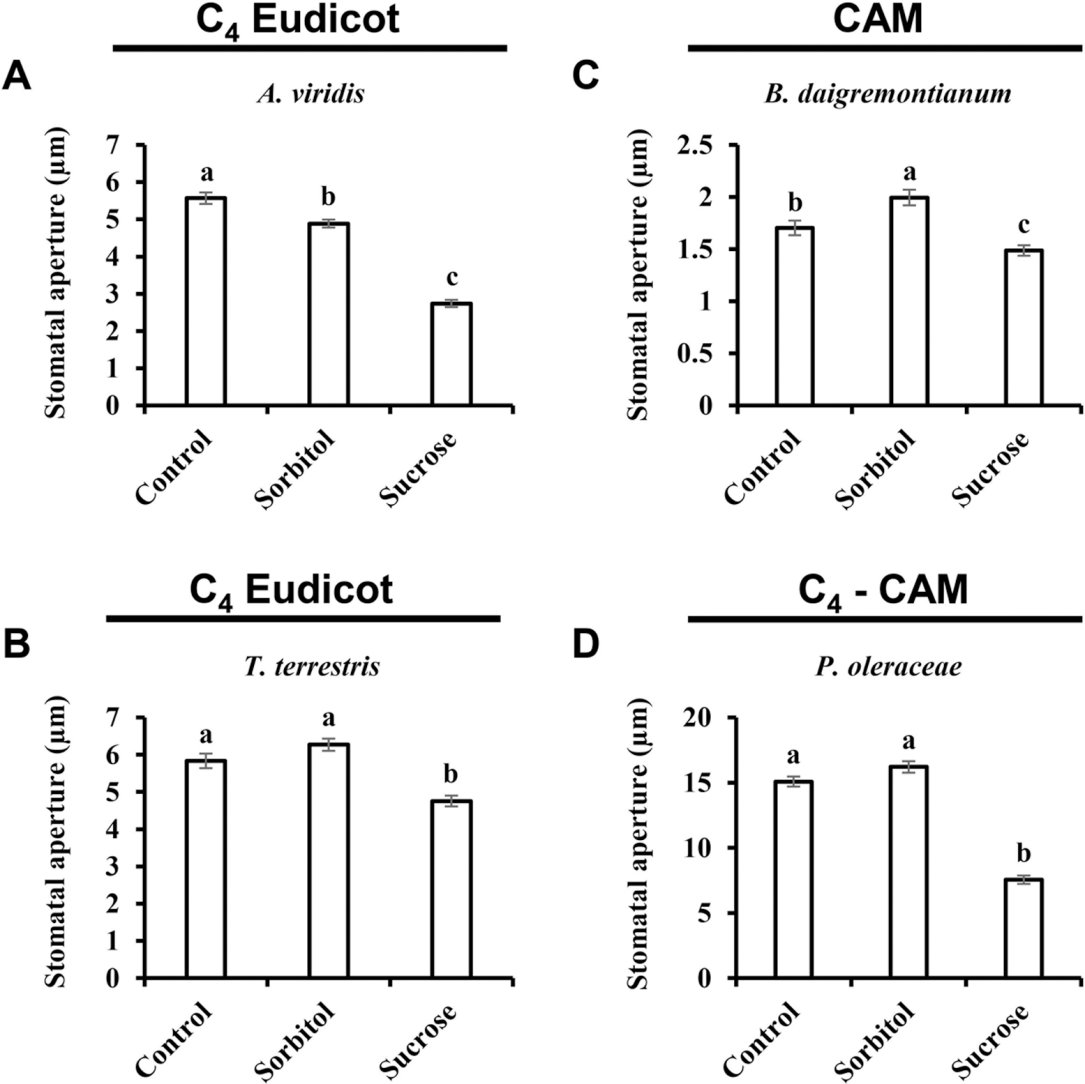
Sucrose-induced stomatal closure in C_4_ and CAM plants. Stomatal response to sucrose was assayed in the C_4_ eudicots **A**) *A*. *viridis* and **B**) *T*. *terrestris*, the CAM plant **C**) *B*. *daigremontianum* and the C_4_-CAM plant **D**) *P*. *oleracea*. This response was assayed by immersing detached leaves for 3 h in artificial apoplastic sap (AS, control) or AS containing 200 mM sorbitol (as an osmotic control) or 200 mM Suc. Data points are means ± SE. [The numbers of independent biological repeats and stomata (*n*) analyzed for each species are listed in S1 Table.] Different letters indicate a significant difference (Tukey's HSD test, *P* < 0.05).

Over the course of evolution, several different photosynthetic strategies have evolved (i.e., C_3_, C_4_, C_3_-C_4_, and CAM). These strategies involve different leaf anatomy and differences in the timing of stomatal opening. Unlike the dispersed mesophyll cells in C_3_ plants, the mesophyll cells of C_4_ plants are arranged in a ring around a bundle-sheath (BS) layer that surrounds the vascular tissue [50]. Sugar production takes place in the BS cells, minimizing the distance between the site of sugar production (in the BS layer) and the phloem-loading site. As a consequence, the responsiveness of the stomata of C_4_ plants to sugars might be different from that of C_3_ plants. As shown above, Suc stimulates stomatal closure of the monocot C_4_ plants *Z*. *mays*, *M*. *paradisiaca* and *S*. *bicolor* (Fig 3A–3C). We also tested two additional eudicot C_4_ species, *Amaranthus viridis* and *Tribulus terrestris*, which belong to the Amaranthaceae and Zygophyllaceae families, respectively (Fig 5A and 5B). Suc triggered stomatal closure in both of those species, decreasing the size of the stomatal apertures of *A*. *viridis* and *T*. *terrestris* by 44% and 24%, respectively (Fig 5A and 5B).

In addition to the spatial separation of mesophyll cells from sugar production found in C_4_ plants, CAM plants also employ temporal separation. The stomata of CAM plants are open at night, during which time malate (C_4_ molecule) is formed using CO_2_, following several enzymatic steps. The C_4_ is stored in the vacuole (as C_4_ malic acid) during the night. During the day, when the stomata of CAM species are closed, the C_4_ molecules move from the vacuole into the chloroplast, where they are decarboxylated, releasing CO_2_, which is fixed to produce sugar [51]. Since sugar is produced in CAM plants when the stomata are closed, we tested whether the stomata of CAM plants are at all responsive to sugar. We initially tested *Bryophyllum daigremontianum* and found that Suc significantly reduced its stomatal closure (25%) relative to Sorb (Fig 5C). Next, we assayed *Portulaca oleracea*, a C_4_-CAM plant that functions as a C_4_ plant under normal conditions and shifts to a CAM strategy under drought conditions [52]. The stomata of *P*. *oleracea* were found to be highly responsive to Suc, which decreased stomatal aperture size by 53% relative to the osmotic control (Fig 5D). Taken together, these results show that Suc induces stomatal closure independent of the photosynthetic strategy used (Fig 5), further indicating that the stomatal response to Suc might be found across a wide range of plant species.

## Discussion

Stomatal opening is essential for CO_2_ uptake for photosynthesis and stomatal closure is necessary to reduce transpiration, maintain plant water potential and prevent desiccation. The coordination between photosynthesis and water loss has been fine-tuned by the evolution of mechanisms that open and close stomata. Passive hydration-dehydration guard-cell movements in ancient primitive plant species have evolved into sophisticated mechanisms activated by physiological and environmental signals, which lead to changes in the osmolyte content of guard cells and stomatal aperture. Light, CO_2_ concentration, vapor pressure deficit, temperature and water availability are among the environmental signals that affect stomatal movement [53, 54]. Yet, a direct effect of the primary product of photosynthesis, sucrose, on stomatal aperture has received little research attention over the years [55]. Red (photosynthetic) light opens stomata and mesophyll cells enhance stomatal opening, indicating that there is a mesophyll-derived product that opens stomata [56, 57]. Over the years, several studies have sought to identify the photosynthetic mesophyll product that opens stomata [56, 58] and sucrose, the obvious mesophyll-exported product, should have been be the primary mesophyll-derived candidate to be considered. Nevertheless, for many years, no functional study reported that sucrose opens stomata and two studies that did report an effect of sucrose found no opening effect and even a closing effect [59, 60]. We assume that those observations regarding the closure effect of sucrose were neglected because they ran counter to the prevailing hypothesis that sucrose is an osmolyte that opens stomata.

It was only recently that a stomatal-closure effect caused by sucrose independent of its extracellular osmotic effect was demonstrated in Arabidopsis, tomato and *V*. *faba* [21–24]. Recent studies examined the response of stomata to different concentrations of sucrose. While low concentrations of sucrose (0.1 and 1 mM) had no effect on stomatal aperture, concentrations of 10 and 100 mM reduced stomatal apertures, supporting the notion that the accumulation of sucrose at the guard cells when photosynthesis rates are high stimulates stomatal closure, thereby coordinating photosynthesis with transpiration [21]. In a multispecies meta-analysis of data from several studies, the relationship between primary metabolism and gas-exchange parameters was modeled and a clear negative correlation was observed between sugar (Suc, glucose and fructose) content and stomatal conductance, further supporting the stomatal-closure effect of sugar [61].

**Fig 6:**
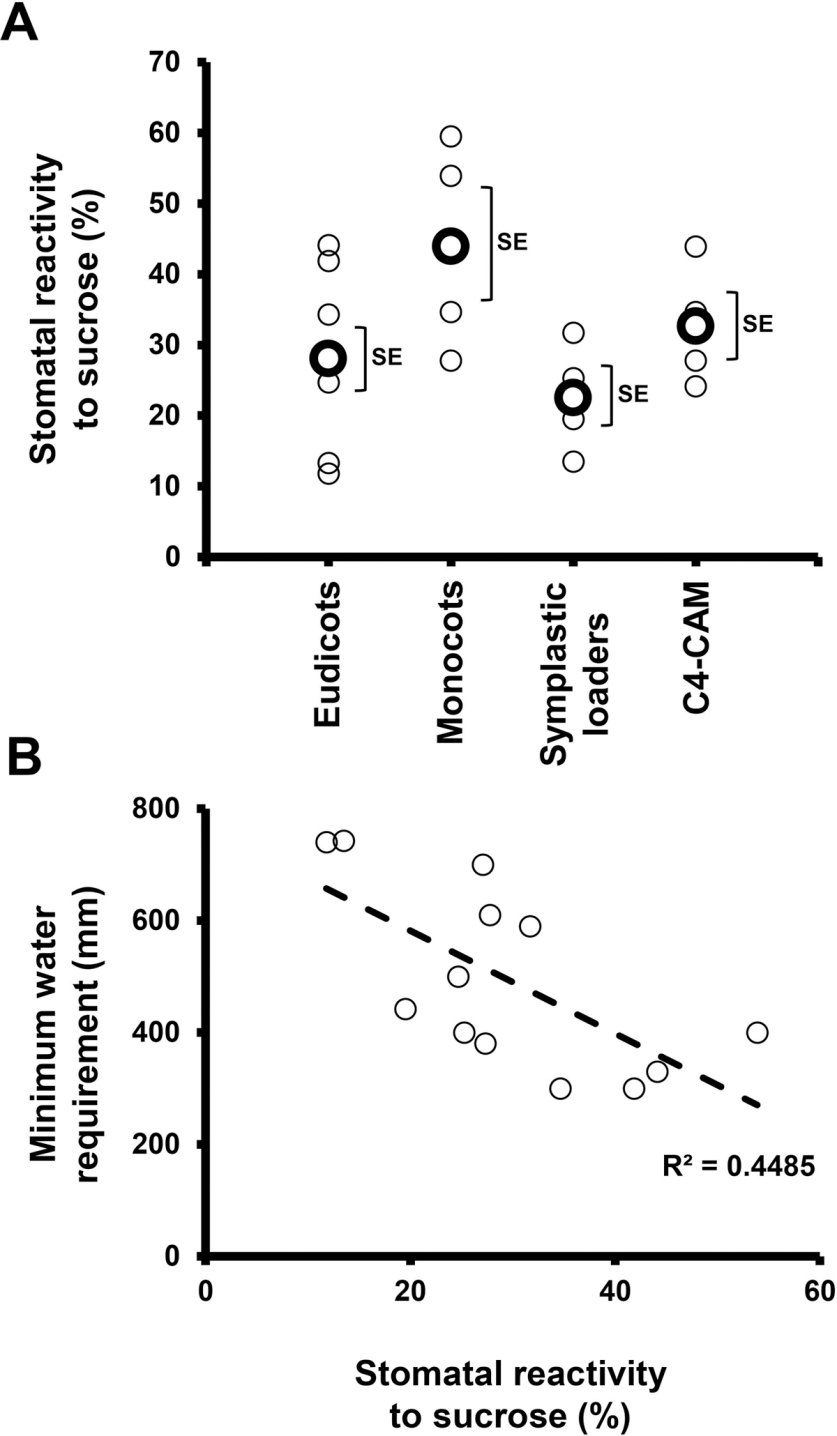
Stomatal reactivity to sucrose across evolution and in correlation with minimum water requirements. (**A**) Stomatal reactivity to sucrose (relative to sorbitol) in the species tested in our survey. Small circles indicate the mean for each species and big, bold circles indicate the mean value for each group (Eudicots, monocots, symplastic loaders and C4-CAM). Brackets indicate the SE. (**B**) A negative correlation was observed between the minimum water requirement (mm) and the stomatal reactivity to sucrose (%).

We assumed that coordination between water loss and sugar production may be a general feature of plants and examined stomatal responses to sucrose in various species representing different evolutionary lineages, photosynthetic systems and phloem-loading strategies. Sucrose appeared to stimulate stomatal closure in most of the examined species (with *O*. *basilicum* being possible exception), regardless of the photosynthetic mechanism (C_3_, C_4_ and CAM) or phloem-loading pathway (symplastic or apoplastic) used. The extent of stomatal closure varied among the different species and may represent species-specific responses that follow no general trends (Fig 6A). Yet, taking a more global perspective, it seems that monocots do display greater stomatal responsiveness to Suc than eudicots. In our study, the monocots presented a responsiveness of about 44% while the average response of the eudicots was about 28% (Fig 6A). It was previously suggested that the overall responsiveness of grass (monocot) stomata to environmental inputs is indeed greater; mainly due to the presence of subsidiary cells adjacent to guard cells, which provide osmolytes for faster responses [8]. Furthermore, this strong sensitivity of grasses is associated with drier climates and habitats in which environmental conditions fluctuate rapidly [8, 62, 63]. Considering this, it appears that stomatal responsiveness to Suc is well correlated with overall stomatal responsiveness. We, therefore, speculate that during evolution, the selection for species that perform better under drought, such as the grasses, occurred in the presence of a need for strict regulation of the relationship between photosynthesis and transpiration, making Suc as a major player in that relationship.

In addition, a slight tendency of heightened guard-cell response to sucrose was observed in species that can survive on lower amounts of water (Fig 6B), suggesting that, under limited-water conditions, a survival advantage may be conferred by a stronger stomatal response to sugar. Such a correlation is notable in the monocots wheat and sorghum, which are generally grown in a semi-arid environments and have minimum water requirements of 400 mm and 300 mm, respectively (Fig 6B), and which displayed enhanced stomatal response to sucrose (54% and 35% for wheat and sorghum, respectively). In contrast, species with tropical origins, such as maize, displayed an opposite pattern of a high water requirement (610 mm) alongside a lower stomatal response to sucrose (28%, Fig 6B). We assume that the stomatal response to sugar is an advantage that has evolved to improve the coordination of photosynthesis and transpiration. In plants with highly sensitive responses, the sensing of high sugar levels leads to enhanced (and perhaps faster) closure of the stomata, in order to preserve more water. These differences in stomatal response could be one of the traits that account for sorghum’s growth advantage over maize under drought conditions [64].

It is easy to envision increasing sucrose levels in the apoplast of apoplastic loaders [20], but sucrose also exists in the apoplast of symplastic loaders [46–49, 65–67]. In addition, guard cells are symplastically isolated and probably obtain mesophyll-produced sugars through the apoplast. Guard cells of various species possess sucrose and glucose transporters, indicating the uptake of apoplastic sugar by guard-cells [28–30]. It is likely that, in symplastic loaders, in addition to the main symplastic route, a certain amount of sucrose is naturally exported to the apoplast to feed non-photosynthetic cells such as the epidermis and guard cells. We assume that this apoplastic sugar also enables the sugar-sensing feedback mechanism leading to stomatal closure that coordinates photosynthesis with transpiration. Nevertheless, compared to the responses observed among the monocots and some of the eudicots, the response in the symplastic loaders was less intense (22%; Fig 6A).

The purpose of this study was to explore how widespread the phenomenon of stomatal closure by sucrose is, rather than to identify the lowest possible concentration of sucrose that can stimulate stomatal closure. When looking for responses to various compounds, it is quite common to use concentrations that might be greater than physiological concentrations (which we believe is not the case here, see below). For example, the discovery of sugar-sensing in plants was done with 6% (~330 mM) glucose in the media, completely unnatural and non-physiological conditions [68, 69]. That is to say that use of non-physiological conditions might be necessary sometimes, in order to observe effects that otherwise might be too small to be noticed. However, we believe that 200 mM is not too far from the sucrose concentrations found under physiological conditions. Overall, there is a very limited knowledge about sugar concentrations in the leaf apoplast, let alone the guard-cell apoplast (perhaps due to technical difficulties involved in collecting such data), except for *V*. *faba*, which accumulates about 150 mM sucrose at its guard-cell apopalst [20]. We assume that under different environmental conditions, such as high light intensity and high VPD, even more sugar might be carried toward and accumulate at the guard cells.

The use of non-metabolic sugars such as sorbitol as an osmotic control for metabolic sugar is very common [21, 23, 70–73]. If sorbitol does not enter the guard cells, it is expected that it will reduce stomatal aperture due to its extracellular osmotic effect. However, if sorbitol does enter the guard cells and reaches a balance with its extracellular concentration, then no effect on stomatal aperture is expected. In several cases, sorbitol did not change the stomatal aperture. But in most cases, sorbitol reduced stomatal apertures, as compared to the non-isosmotic control (artificial sap–AS), probably due to its extracellular osmotic effect. However, in some cases, sorbitol increased stomatal aperture relative to the control (Figs 1A, 2H, 4C and 5C). We assume that, in those cases, sorbitol entered and perhaps accumulated in guard cells, thereby contributing to the guard cells’ osmotic potential and opening stomata. Yet, sucrose or its cleavage products glucose and fructose also enter the guard cells via sucrose and hexose transporters [28–30] and may contribute to guard-cell osmotic potential. But unlike sorbitol that opens stomata in few cases, sucrose closes stomata, supporting a sugar-closure effect.

The results of this study show that stomatal closure by sucrose (observed in all of the species examined in this study (with the possible exception of basil) might have evolved early in evolution and been preserved in divergent plant species that use different photosynthetic and sugar-transport strategies. Whether it also exists in earlier lineages such as pteridophytes and bryophytes, which might employ a hydration-dehydration passive stomatal response, remains to be determined. Nevertheless, stomatal response to sucrose might be a universal response aimed at coordinating sugar production with water loss.

## Supporting information

S1 Table Numbers of independent biological repeats (leaves) and stomatal repetitions (*n*) for each species and treatment. (DOCX)
