# Supplementary material for: Sucrose-induced stomatal closure is conserved across evolution
Source: PLoS One. 2018 Oct 12;13(10):e0205359. doi: 10.1371/journal.pone.0205359 (PMC6185732; doi:10.1371/journal.pone.0205359)
Supplement: S1 Table — (DOCX) [file pone.0205359.s001.docx]

|  | **Species** | **AS** | | **AS + Sorbitol** | | **AS + Sucrose** | |  |
| --- | --- | --- | --- | --- | --- | --- | --- | --- |
|  | | **No. of leaves** | **No. of stomata (*n*)** | **No. of leaves** | **No. of stomata (*n*)** | **No. of leaves** | **No. of stomata (*n*)** |  |
| **Fig. 1** | *Triticum aestivum* L. | 3 | 161 | 100 mM- 4  200 mM- 3 | 100 mM- 180  200 mM- 148 | 100 mM- 3  200 mM- 3 | 100 mM- 168  200 mM- 158 |  |
|  | *Citrullus lanatus* (Thunb.) Matsum. & Nakai | 3 | 157 | 100 mM- 3  200 mM- 3 | 100 mM- 151  200 mM- 164 | 100 mM- 3  200 mM- 3 | 100 mM- 165  200 mM- 169 |  |
|  | | | | | | | |  |
| **Fig. 2** | *Ricinus communis* L. | 4 | 201 | 3 | 164 | 4 | 182 |  |
|  | *Populus angulata* Aiton. | 4 | 249 | 4 | 172 | 4 | 161 |  |
|  | *Oxalis corniculata* L. | 3 | 130 | 5 | 206 | 4 | 146 |  |
|  | *Vitis vinifera* L. | 5 | 350 | 5 | 299 | 5 | 360 |  |
|  | *Catharanthus roseus* (L.) G. Don | 4 | 194 | 4 | 183 | 3 | 144 |  |
|  | *Pelargonium hortorum* L.H. Bailey | 4 | 199 | 4 | 200 | 4 | 171 |  |
|  | *Melia azadirachta* L. | 5 | 334 | 4 | 236 | 4 | 208 |  |
|  | *Moringa oleifera* Lam. | 5 | 374 | 5 | 470 | 5 | 394 |  |
|  | | | | | | | |  |
| **Fig. 3** | *Zea mays* L. | 5 | 198 | 5 | 188 | 5 | 195 |  |
|  | *Sorghum bicolor* (L.) Moench | 4 | 193 | 4 | 129 | 5 | 132 |  |
|  | *Musa × paradisiaca* L*.* | 4 | 200 | 4 | 146 | 4 | 130 |  |
|  | | | | | | | |  |
| **Fig. 4** | *Cucumis melo* L. | 3 | 133 | 3 | 145 | 3 | 137 |  |
|  | *Cucurbita pepo* L. var. *cylindrica* | 4 | 384 | 4 | 396 | 4 | 390 |  |
|  | *Ocimum basilicum* L. | 6 | 270 | 5 | 234 | 5 | 196 |  |
|  | | | | | | | |  |
| **Fig. 5** | *Amaranthus viridis* L*.* | 4 | 209 | 4 | 242 | 4 | 165 |  |
|  | *Tribulus terrestris* L. | 3 | 72 | 4 | 142 | 4 | 195 |  |
|  | *Bryophyllum daigremontianum* (Raym.-Hamet & H. Perrier) A. Berger | 5 | 172 | 5 | 186 | 5 | 180 |  |
|  | *Portulaca oleracea* L. | 4 | 170 | 3 | 114 | 4 | 146 |  |
|  | | | | | | | |  |

**S1 Table**

Numbers of independent biological repeats (leaves) and stomatal repetitions (*n*) for each species and treatment
